# Presenting comparative study PRO results to clinicians and researchers: beyond the eye of the beholder

**DOI:** 10.1007/s11136-017-1710-6

**Published:** 2017-11-02

**Authors:** Michael Brundage, Amanda Blackford, Elliott Tolbert, Katherine Smith, Elissa Bantug, Claire Snyder, PRO Data Presentation Stakeholder Advisory Board (various names and locations), Neil K. Aaronson, Patricia A. Ganz, Ravin Garg, Michael Fisch, Vanessa Hoffman, Bryce B. Reeve, Eden Stotsky-Himelfarb, Ellen Stovall, Matthew Zachary

**Affiliations:** 1 Department of Oncology, Queens Cancer Research Institute, Queen’s University Kingston, Kingston, ON Canada; 2 Johns Hopkins School of Medicine, 550 N. Broadway, Room 1111, Baltimore, MD 21205 USA; 3 Johns Hopkins School of Medicine, Baltimore, MD USA; 4 Johns Hopkins Bloomberg School of Public Health, 624 N. Broadway, Room 725, Baltimore, MD 21205 USA; 5 Johns Hopkins Bloomberg School of Public Health, 624 N. Broadway, Room 726, Baltimore, MD 21205 USA; 6 Johns Hopkins Bloomberg School of Public Health, 624 N. Broadway, Room 649, Baltimore, MD 21205 USA; 7 Sidney Kimmel Comprehensive Cancer Center at Johns Hopkins, 1650 Orleans Street, Baltimore, MD 21287 USA; 8 Cancer Clinic of Southeastern Ontario, 25 King Street West, Kingston, ON K7L 5P9 Canada

**Keywords:** Patient-reported outcomes, Graphic communication, Knowledge translation, Clinical trials, Comparative effectiveness research

## Abstract

**Purpose:**

Patient-reported outcome (PRO) results from clinical trials can inform clinical care, but PRO interpretation is challenging. We evaluated the interpretation accuracy and perceived clarity of various strategies for displaying clinical trial PRO findings.

**Methods:**

We conducted an e-survey of oncology clinicians and PRO researchers (supplemented by one-on-one clinician interviews) that randomized respondents to view one of the three line-graph formats (average scores over time for two treatments on four domains): (1) higher scores consistently indicating “better” patient status; (2) higher scores indicating “more” of what was being measured (better for function, worse for symptoms); or (3) normed scores. Two formats displayed proportions changed (pie/bar charts). Multivariate modeling was used to analyze interpretation accuracy and clarity ratings.

**Results:**

Two hundred and thirty-three clinicians and 248 researchers responded; ten clinicians were interviewed. Line graphs with “better” directionality were more likely to be interpreted accurately than “normed” line graphs (OR 1.55; 95% CI 1.01–2.38; *p* = 0.04). No significant differences were found between “better” and “more” formats. “Better” formatted graphs were also more likely to be rated “very clear” versus “normed” formatted graphs (OR 1.91; 95% CI 1.44–2.54; *p* < 0.001). For proportions changed, respondents were less likely to make an interpretation error with pie versus bar charts (OR 0.35; 95% CI 0.2–0.6; *p* < 0.001); clarity ratings did not differ between formats. Qualitative findings informed the interpretation of the survey findings.

**Conclusions:**

Graphic formats for presenting PRO data differ in how accurately they are interpreted and how clear they are perceived to be. These findings will inform the development of best practices for optimally reporting PRO findings.

**Electronic supplementary material:**

The online version of this article (doi:10.1007/s11136-017-1710-6) contains supplementary material, which is available to authorized users.

## Introduction

An emphasis on patient-centered care has increased the demand for patient-reported outcomes (PROs), data collected directly from patients about health conditions and impacts of treatments [1, 2]. PROs can inform patient care in a variety of ways [3, 4], including data representing ‘the voice of the patient’ in randomized clinical trials to inform decision-making by patients and clinicians based on trial results [4–7]. Oncologists have endorsed the use of PROs for this purpose, and there is evidence that PRO results can influence treatment choices [8–11].

The optimal integration of PRO results from clinical trials, and other comparative research into clinical care requires that clinicians understand and interpret PROs accurately and be able to communicate PRO findings to their patients where appropriate. This understanding can be challenging because of the variety of PRO questionnaires, variation in their scoring (e.g., higher scores indicating better or worse outcomes), their scaling (e.g., scores ranging from 0 to 100 as worst-to-best, or scores normed to a defined population), and how statistical and clinical significance of the findings are addressed [9, 12–15].

This study was part of a larger research program designed to examine approaches for presenting PRO data to promote their understanding and use. Previously, we evaluated existing approaches to presenting study PRO results [15]. We then partnered with stakeholder workgroups of clinicians and patients to develop improved graphical presentation approaches [14]. In a separate research stream, we focused on the communication of PROs to patients (e.g., in educational materials or decision aids) [16]. In this study, we focused on the reporting of PROs from clinical trials to clinicians and PRO researchers, building on our earlier study findings that identified the visual presentation strategies with the greatest potential for effective data communication [14, 15]. In the context of a clinical trial randomizing patients to one of the two treatment groups, we addressed between-group comparisons of mean scores over time, and between-group comparisons of the proportions of patients changed from baseline (improved, stable, or worsened). Our objective was to evaluate interpretation accuracy, clarity, and preferences for these candidate presentation approaches in a broad population of clinicians and PRO researchers.

## Methods

### Study design

We conducted a cross-sectional, mixed-methods study comprising a survey of cancer clinicians and PRO researchers, complemented by qualitative interviews with clinicians. We used an internet-based survey to evaluate how clinician and PRO researcher respondents interpret graphically displayed PRO results from a hypothetical clinical trial comparing Treatments “X” and “Y”. We supplemented the survey findings by administering it to cancer clinicians using one-on-one interviews, thus obtaining qualitative data on their responses. As the focus of the project was on clinicians’ understanding and communication of PRO data in clinical practice, we did not undertake on-one-one interviews with PRO researchers. The Johns Hopkins School of Medicine Institutional Review Board approved the project. The funding source had no role in study design, data collection, analysis, interpretation, writing, or publication decision.

### Population and setting

We invited oncology clinicians and PRO researchers to complete the online survey by recruiting a convenience sample using a “snow-ball” approach. We partnered with our study Stakeholder Advisory Board to distribute the survey link to a variety of target populations in order to achieve diversity among respondents. Recipients were encouraged to share the information with other individuals who fit the eligibility criteria. Survey eligibility was self-identification as a health care provider to cancer patients, or PRO researcher.

Respondents who completed the one-on-one interviews were recruited from the Johns Hopkins Clinical Research Network (JHCRN), a consortium of academic and community health systems in Maryland, Virginia, and the District of Columbia (USA). Eligible clinicians were active oncologists (medical, radiation, surgical, gynecologic/urologic), nurse practitioners/physician assistants, or fellows. Purposive sampling was done to include various specialties and JHCRN sites.

### Study procedures

**Fig. 1:**
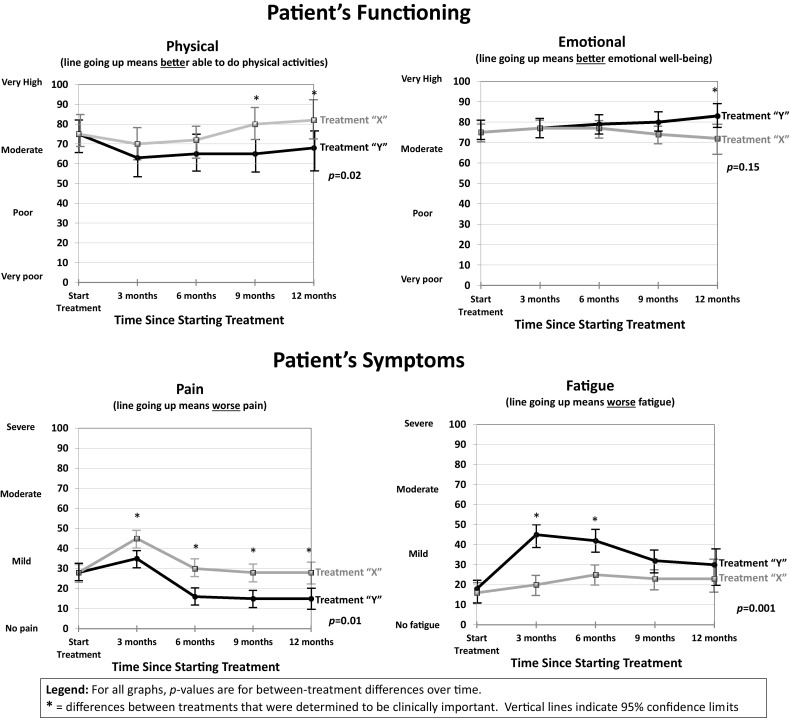
Example illustrating average group scores over time, formatted for higher scores indicating “more” of what was being measured. The example shows the “confidence limit” annotation variation used across format types

The online survey randomized each eligible respondent to one of the eighteen survey versions. Each version sequentially presented five formats for graphically presenting clinical trial results comparing two treatments. Specifically, three line-graph format variations showing average group scores over time were displayed, followed by two formats showing proportions changed (better/worse/about the same) at 9 months after initiating treatment. The formats were chosen based on our earlier research that identified the most promising formats for effective PRO data communication [14, 15]. For each format, results for four PRO domains (two functions, two symptoms) were displayed on a single screen (Fig. 1).

**Fig. 2:**
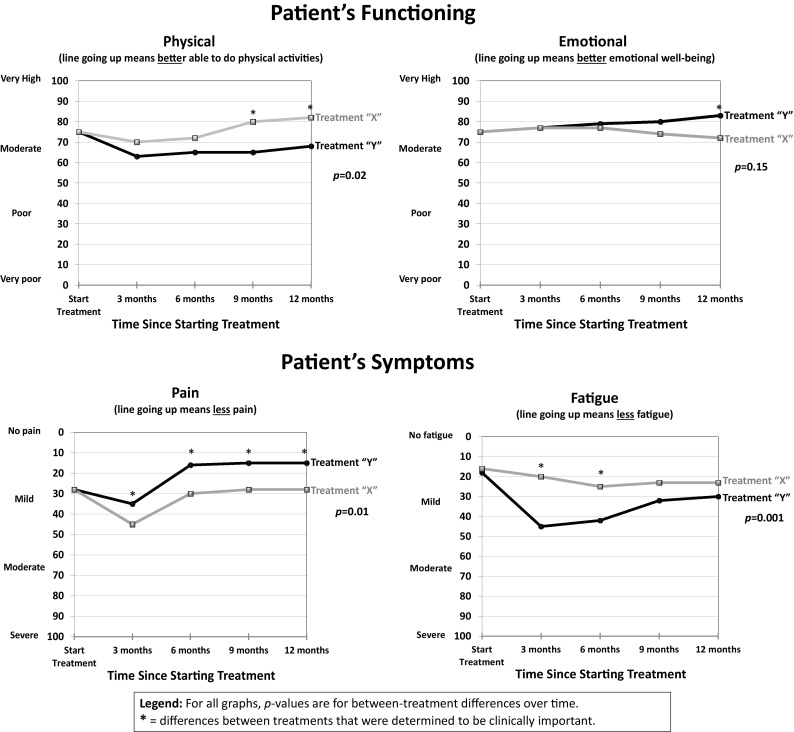
Example of average group scores over time, formatted for higher scores indicating “better” outcomes. The example shows the “clinical significance” annotation variation used across format types

**Fig. 3:**
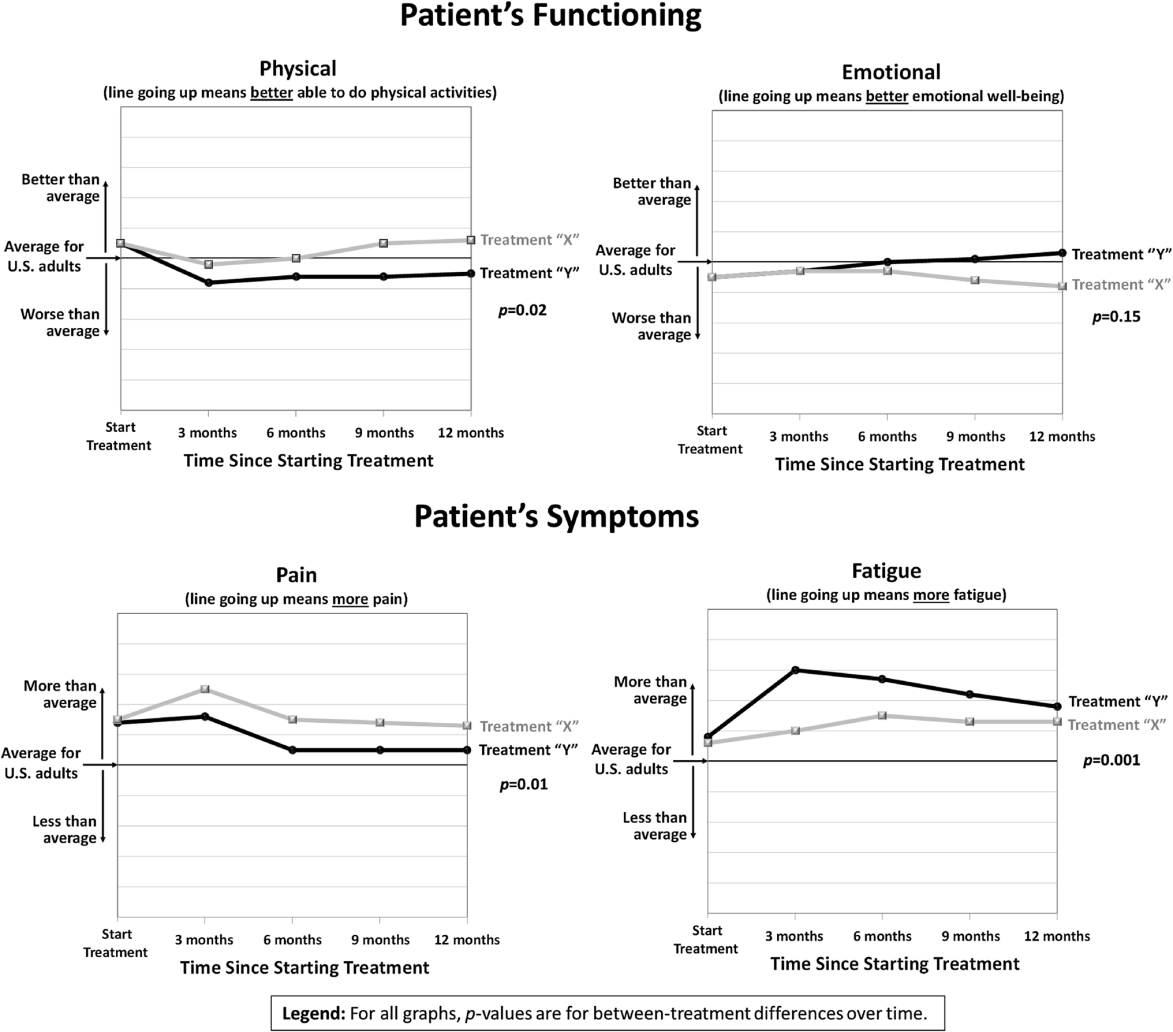
Example of average group scores over time, formatted to be normed to the general U.S. population. The example shows the “plain” variation (no additional annotations) used across format types

**Fig. 4:**
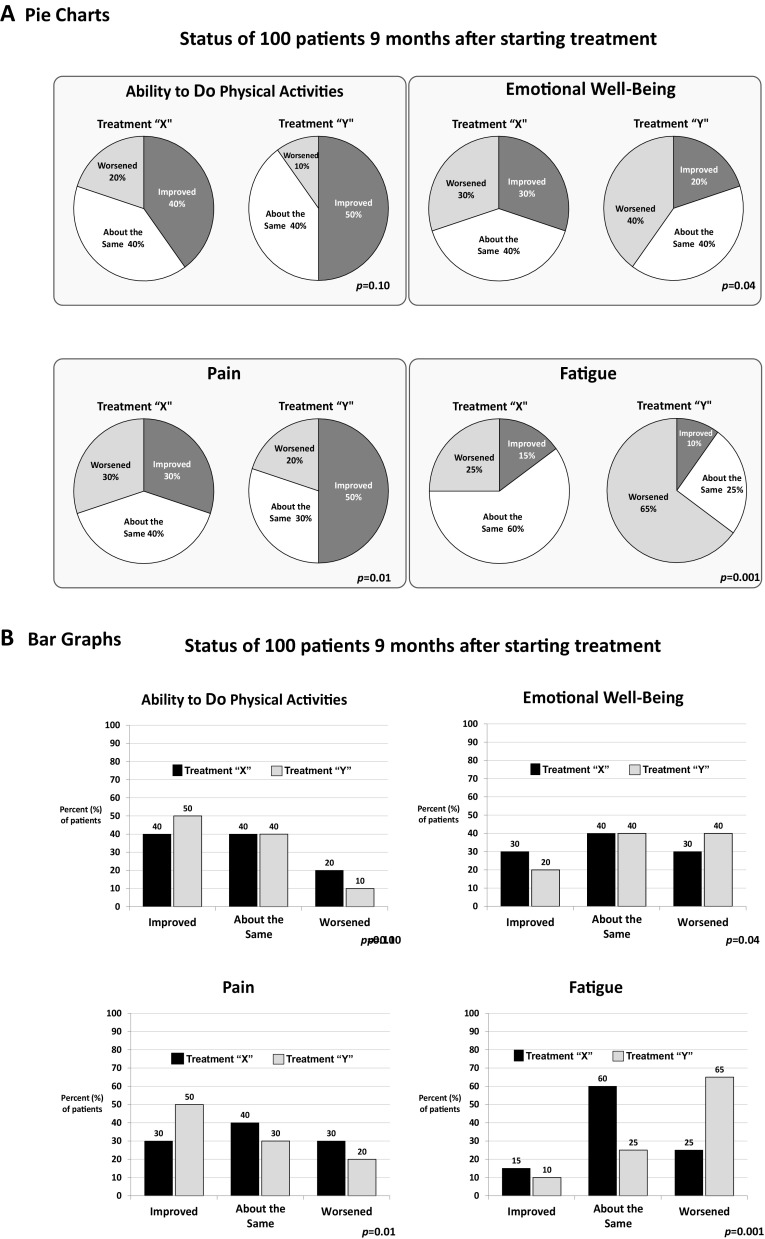
Examples of formats illustrating the proportions of patients changed at 9 months (compared to baseline). **a** Pie chart format. **b** Bar chart format

Respondents were randomized to one of the three line-graph format types: one-third of the sample saw graphs wherein PRO results indicated “more” of what was being measured (e.g., as per the EORTC QLQ-C30 [17].): a line trending up for functional domains indicated improvement, whereas a line trending up for symptom domains indicated worsening (more symptoms, Fig. 1). For one-third of respondents, lines trending up consistently indicated improvement (“better”) outcomes for both function and symptom domains (e.g., the HUI [18] Fig. 2). The remaining one-third viewed data normed to a general population average of 50 (Fig. 3). Within each line-graph type (“more,” “better,” “normed”), three variations were displayed: “plain” lines (*p* values only, Fig. 3); clinically significant between-arm differences indicated by an asterisk (but no confidence limits, Fig. 2); and confidence limits in addition to the asterisk indicating clinical significance (Fig. 1). Following presentation of line graphs, respondents viewed formats illustrating the proportions of patients either improved, stable, or worsened at 9 months (as typically reported in RCTs by classifying each patient as improved or worsened, compared to baseline, as defined by a clinically important difference established for the PRO measure used in the trial). Respondents viewed these proportions as both pie charts and bar charts (Fig. 4). In sum, all respondents saw one type of line graph (“more,” “normed,” or “better” scaling), presented in three variations (“plain,” “clinical significance,” and “confidence limits”) followed by two proportions changed formats.

We controlled for potential order effects by randomizing respondents to order of line-graph variation and order of proportion format display. Supplemental Table S1 summarizes the formats and orders used across survey versions. The underlying hypothetical clinical trial results were the same across orders.

Study outcomes included respondents’ accuracy of interpreting between-group differences, their ratings of format clarity, and their preferred format. Two accuracy questions were asked on the first line-graph format seen (one for a function and one for a symptom domain). For example, for Fig. 1 we asked “On which treatment do patients report better PHYSICAL function over time?” (Response options: “Treatment ‘X,’ Treatment ‘Y,’ Treatments are about the same”). The third response option was coded as “correct” if the *p* value was not significant. One accuracy question was asked on the second and third line-graph format seen. This design enabled us to compare the accuracy of interpretation across the line types, and to test if accuracy rates were associated with added clinical and statistical significance annotations. For the two formats showing proportions changed, two accuracy questions were asked on each (e.g., “At 9 months, on which treatment did more patients improve with regard to PHYSICAL activities?”; same response categories).

Upon completion of accuracy questions for each format, respondents rated its clarity (“very confusing,” “somewhat confusing,” “somewhat clear,” or “very clear”). An open-ended text box allowed comments. After viewing all five formats, respondents were asked to select the proportion they thought was most useful for showing trial results, and which line-graph variation was most useful. Upon conclusion, respondents could enter for a chance to receive a $100 Amazon gift card.

One-on-one interview respondents were randomly assigned to complete one of the online survey versions. Respondents were asked to think aloud as they completed the survey. The interviewer prompted for comments concerning a format’s clarity or about preferences for particular format. At the end of the interview, respondents were given an opportunity to share any overall feedback that was not captured during survey completion.

### Analysis

Quantitative data included respondents’ demographic characteristics, their accuracy and clarity responses, interpretations of statistical and clinical significance, and format preferences. These data were first summarized descriptively. We used multivariable generalized estimating equation (GEE) logistic regression models (with the individual as the cluster unit) to test differences in accuracy and clarity by format while controlling for format order and respondent type. Two outcomes were used to evaluate interpretation accuracy: (1) accuracy on the two questions for the first format seen and (2) accuracy for the four questions on each format across all orders (and, therefore, all respondents). Fixed effects for the specific questions were included in the model that included all questions. For calculating accuracy rates, we coded responses as “correct” when they matched the intended response. Given the potential ambiguity in how respondents selected the “treatments are about the same” response, we also calculated the proportion of respondents that responded incorrectly in absolute terms (i.e., picking Treatment “X” when “Y” was correct).

Qualitative data obtained from the one-on-one interviews were analyzed by a coding scheme based on the study objectives, interview structure, and interview content. Codes were created to capture positive or negative comments, misinterpretations, directional concerns, preferences, and other comments. One member of the research team coded each transcript using ATLAS.ti, [19] a second member reviewed coding, and a report was then generated to identify themes. Open-ended text box comments obtained from online survey respondents were sorted into broad, preexisting categories of “positive” and “negative.”

## Results

### Study sample

The internet sample included 233 clinicians and 248 PRO researchers (total *N* = 481) (Table 1). Clinicians reported an average time in practice of 16.5 years, 55% practiced medical oncology, and half were located in the US. Among PRO researchers, 38% had > 10 years’ experience, and 43% were from the US. The in-person interviewees included ten clinicians: one medical oncologist, one radiation oncologist, one urologist, one oncology nurse practitioner, three surgical oncologists, and three oncology fellows; five were from Johns Hopkins.

**Table 1:** Participant characteristics

Characteristic	Clinicians (*N* = 233)		Researchers (*N* = 248)	
Age (median, range)	41	(25–78)	43	(23–73)
Years in practice (average, std. deviation)	16.5	(12.5)	–	–

	*N*	(%)	*N*	(%)
Gender				
Female	108	(54.8)	131	(63.3)
Male	89	(45.2)	76	(36.7)
Missing	36		41	
Race				
White	145	(73.6)	162	(78.6)
Black	3	(1.5)	5	(2.4)
Asian	35	(17.8)	29	(14.1)
Other	14	(7.1)	10	(4.9)
Missing	36		42	
Country				
USA	98	(50.0)	87	(43.1)
Netherlands	0	0	12	(5.9)
UK	6	(3.1)	10	(5.0)
Canada	6	(3.1)	16	(7.9)
Other	86	(43.9)	77	(38.1)
Missing	37		46	
Clinician specialty				
Medical oncology	108	(54.5)	–	–
Radiation oncology	13	(6.6)		
Surgical/gynecologic/urologic oncology	17	(8.6)		
Oncology nurse practitioner or assistant	10	(5.1)		
Other	50	(25.3)		
Missing	35			
Researcher expertise (more than one may apply)				
Patient perspective	–	–	22	(8.9)
Clinician			26	(10.5)
Clinician scientist			60	(24.2)
PRO assessment/psychology/sociology			93	(37.5)
Clinical trials methods/analysis			52	(21.0)
Psychometrics			55	(22.2)
Health policy or public health			37	(14.9)
Journal editor			8	(3.2)
Frequent journal reviewer			52	(21.0)
Regulator or administrator			8	(3.2)
Other			23	(9.3)
PRO research experience				
Current student	–	–	23	(11.1)
Current post-doc			17	(8.2)
< 5 years’ experience			39	(18.8)
5–10 years’ experience			50	(24.2)
> 10 years’ experience			78	(37.7)
Missing			41	

### Findings for line-graph formats

**Fig. 5:**
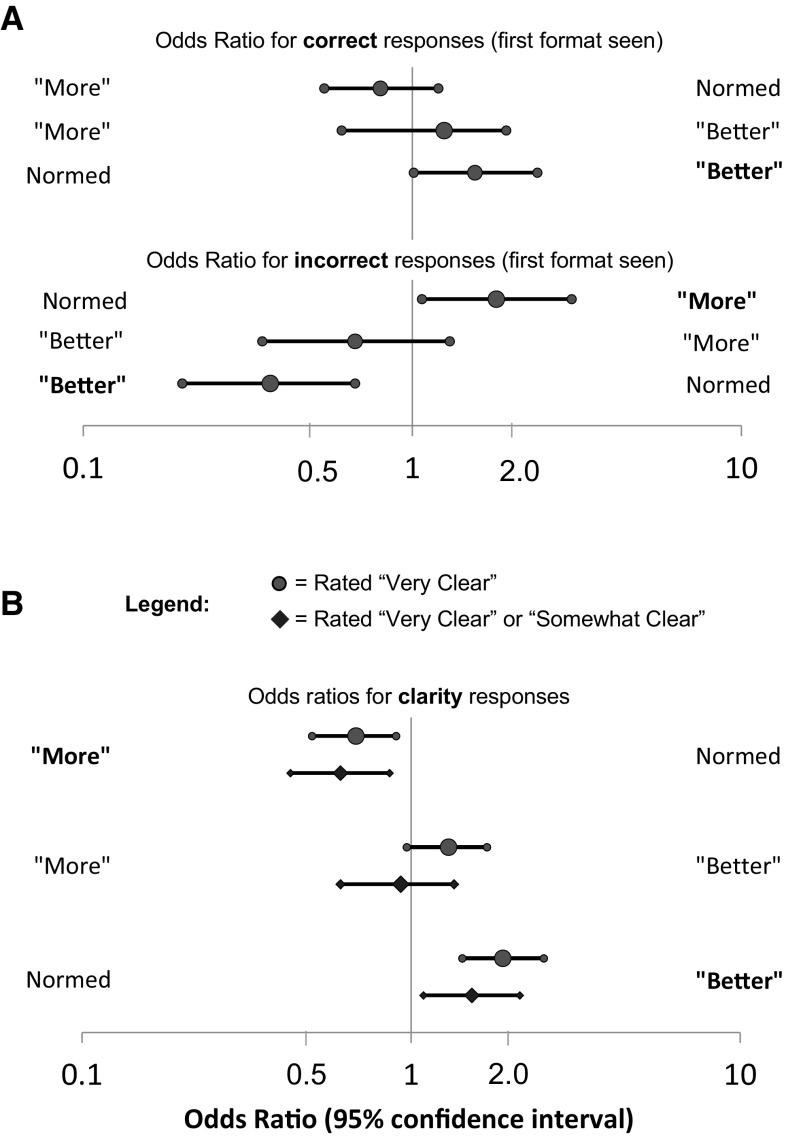
Multivariable modeling results displayed as odds ratios (ORs) for accuracy and clarity ratings for line-graph formats. The point-estimate of each OR indicates the preferred comparitor (format more accurately interpreted or more clearly rated). Significant findings are displayed in bold-face

Participants’ accuracy of interpretations varied across the three line-graph types: clinicians were more likely to answer both initial accuracy questions correctly if they saw the “better” line graphs (68%), versus 62% for “more” line graphs and 61% for “normed” line graphs. The same was true for researchers: 68% correct for “better,” versus 64% for “more,” and 54% for “normed.” Complete descriptions of the responses by format can be found in Table 2. Multivariate model results (Table 3) indicate that “better” line graphs were significantly more likely to be interpreted accurately vs. “normed” (OR 1.55; 95% CI 1.01–2.38; *p* = 0.04). With regard to “incorrect” responses, respondents were significantly more likely to select an incorrect response for “normed” graphs compared to “better” or “more” when controlling for other factors (Table 3). The odds ratios are shown graphically in Fig. 5.

**Table 2:** Accuracy and clarity responses for “line graphs of average scores” formats

	Clinicians			Researchers		
	“More” line type *N* = 78	“Normed” line type *N* = 77	“Better” line type *N* = 78	“More” line type *N* = 83	“Normed” line type *N* = 83	“Better” line type *N* = 82
	*N* (%)	*N* (%)	*N* (%)	*N* (%)	*N* (%)	*N* (%)
Accuracy of interpretation						
Physical activities (function)						
Treatment X^a^	60 (76.9)	61 (79.2)	61 (78.2)	72 (86.7)	71 (85.5)	66 (80.5)
Treatment Y	0	4 (5.2)	2 (2.6)	3 (3.6)	1 (1.2)	2 (2.4)
About the same	8 (10.3)	5 (6.5)	11 (14.1)	5 (6.0)	5 (6.0)	8 (9.8)
Missing	10 (12.8)	7 (9.1)	4 (5.1)	3 (3.6)	6 (7.2)	6 (7.3)
Pain (symptom domain)						
Treatment X	9 (11.5)	13 (16.9)	7 (9.0)	13 (15.7)	23 (27.7)	6 (7.3)
Treatment Y^a^	53 (67.9)	50 (64.9)	64 (82.1)	59 (17.1)	48 (57.8)	62 (75.6)
About the same	3 (3.8)	6 (7.8)	3 (3.8)	5 (6)	5 (6.0)	6 (7.3)
Missing	13 (16.7)	8 (10.4)	4 (5.1)	6 (7.2)	7 (8.4)	8 (9.8)
Number of questions “correct”						
Both questions	48 (61.5)	47 (61.0)	53 (67.9)	53 (63.9)	45 (54.2)	56 (68.3)
One question	17 (21.8)	17 (22.1)	19 (24.4)	25 (30.1)	29 (34.9)	16 (19.5)
Neither question	13 (16.7)	13 (16.9)	6 (7.7)	5 (6)	9 (10.8)	10 (12.2)
Number of questions “incorrect”^b^						
Both questions	0	0	0	0	0	0
One question	9 (11.5)	17 (22.1)	9 (11.5)	16 (19.3)	24 (28.9)	8 (9.8)
Neither question	69 (88.5)	60 (77.9)	69 (88.5)	67 (80.7)	59 (71.1)	74 (90.2)
Clarity ratings: plain line graphs						
Very clear	29 (46.8)	17 (25.4)	32 (44.4)	28 (37.3)	28 (37.3)	34 (48.6)
Somewhat clear	23 (37.1)	34 (50.7)	28 (38.9)	36 (48.0)	32 (42.7)	16 (22.9)
Somewhat confusing	10 (16.1)	15 (22.4)	11 (15.3)	11 (14.7)	12 (16.0)	18 (25.7)
Very confusing	0	1 (1.5)	1 (1.4)	0	3 (4)	2 (2.9)
Missing	16	10	6	8	8	12
Clarity ratings: lines with confidence limits						
Very clear	20 (31.2)	15 (22.1)	30 (41.1)	24 (32.9)	22 (29.3)	32 (47.8)
Somewhat clear	28 (43.8)	30 (44.1)	29 (39.7)	31 (42.5)	28 (37.3)	24 (35.8)
Somewhat confusing	15 (23.4)	22 (32.4)	10 (13.7)	16 (21.9)	20 (26.7)	8 (11.9)
Very confusing	1 (1.6)	1 (1.5)	4 (5.5)	2 (2.7)	5 (6.7)	3 (4.5)
Missing	14	9	5	10	8	15
Clarity ratings: lines with clinical significance						
Very clear	29 (46.8)	22 (32.8)	34 (47.2)	35 (47.9)	28 (37.8)	32 (47.1)
Somewhat clear	28 (45.2)	34 (50.7)	27 (37.5)	30 (41.1)	32 (43.2)	30 (44.1)
Somewhat confusing	5 (8.1)	10 (14.9)	10 (13.9)	7 (9.6)	13 (17.6)	5 (7.4)
Very confusing	0	1 (1.5)	1 (1.4)	1 (1.4)	1 (1.4)	1 (1.5)
Missing	16	10	6	10	9	14

^a^Denotes “correct” response

^b^Answers were coded as incorrect when the wrong treatment was selected by the respondent (answers to “about the same” response were ignored—see text for further details)

**Table 3:** Multivariate modeling results for accuracy of interpretation and clarity ratings

Comparison	Accuracy of interpretation				Format clarity ratings			
	Correct response		Incorrect response		Rated “somewhat” or “very” clear		Rated “very” clear	
	OR [95% CI]	*p*	OR [95% CI]	*p*	OR [95% CI]	*p*	OR [95% CI]	*p*
Model for line-graph formats^a^								
Normed v. “More”	0.80 [0.54–1.21]	.30	**1.81 [1.07, 3.05]**	**.03**	**0.61 [0.43–0.86]**	**.005**	**0.66 [0.50–0.88]**	**.005**
“Better” v. “More”	1.25 [0.81–1.93]	.31	0.67 [0.35, 1.27]	.22	0.93 [0.65–1.35]	.72	1.27 [0.96–1.67]	.09
“Better” vs. normed	**1.55 [1.01–2.38]**	**.04**	**0.37 [0.2, 0.67]**	**.001**	**1.53 [1.09–2.14]**	**.01**	**1.91 [1.44–2.54]**	**< .001**
Confidence limits vs. plain	0.71 [0.47, 1.07]	.10	0.84 [0.48, 1.47]	.53	0.73 [0.53, 1.01]	.06	0.78 [0.59, 1.03]	.08
Asterisks vs. plain	1.01 [0.66, 1.55]	.95	0.99 [0.57, 1.69]	.96	**1.64 [1.13, 2.38]**	**.01**	1.15 [0.87, 1.52]	.31
Confidence limits vs. asterisks	0.7 [0.46, 1.06]	.09	0.85 [0.48, 1.49]	.57	**0.44 [0.31, 0.63]**	**< .001**	**0.67 [0.51, 0.89]**	**.006**
Clinicians vs. researchers	0.95 [0.67, 1.34]	.77	0.75 [0.48, 1.19]	.22	1.02 [0.77, 1.35]	.92	0.87 [0.69, 1.1]	.24
Model for proportions changed formats^b^								
Pie charts vs. bar graphs	1.0 [0.85, 1.19]	.96	**0.35 [0.2, 0.6]**	**< .001**	1.12 [0.83, 1.51]	.47	1.2 [0.91, 1.59]	.20
Clinicians vs. researchers	1.06 [0.78, 1.44]	.73	1.48 [0.82, 2.67]	.20	1.02 [0.75, 1.39]	.90	1.12 [0.84, 1.49]	.44

^a^Adjusted odds ratios from a single multivariable logistic regression model estimated using generalized estimating equations [GEE] with the cluster unit as the individual respondent. Terms for the fixed effects of the specific question were included in the models for accuracy. Adjusted for respondent type [clinician, researcher] and for whether line-graph formats were seen before or after proportions changed formats. Statistically significant findings are in bold font

^b^Adjusted odds ratios from a single multivariable logistic regression model estimated using generalized estimating equations [GEE], with the cluster unit as the individual respondent. Terms for the fixed effects of the specific question were included in the models for accuracy. Adjusted for respondent type [clinician, researcher] and for whether proportions formats were seen before or after line graphs. Statistically significant findings are in bold font

The clarity ratings for clinicians and researchers randomized to “more,” “better,” or “normed” line graphs also varied by format type. The multivariate models indicated that “normed” versions were less likely to be rated “very” clear (OR 0.66; 95% CI 0.50–0.88; *p* = 0.005) compared to “more” types (Table 3). Further, the “better” line types were more likely to be rated “very” clear compared to “normed” (OR 1.91; 95% CI 1.44–2.54; *p* < 0.0001), whereas ratings for “more” vs “better” line type did not differ significantly. Similar patterns were seen for combined “somewhat” and “very” clear rating categories. These data are described in full in Table 2 and displayed graphically in Fig. 5.

Across clinicians and researchers, and regardless of randomization to “better,” “more,” or “normed” versions, the format variations with asterisks indicating clinical significance and with confidence limits were not associated significantly with accuracy of interpretations compared to “plain” line-graph formats (Table 3). The variation with only clinical significance indicated was most likely to be rated “somewhat” or “very” clear, compared to the plain or confidence limit variations (Table 3). With regard to individuals’ expressed preferences, both clinicians and researchers preferred the confidence limit variation (52 and 49%, respectively; both *p* < 0.001, Table 4).

**Table 4:** Preferred format: lines (by line format and responder group)

	Clinicians				Researchers			
	Regular *N* = 78	Normed *N* = 77	Reversed *N* = 78	All^a^	Regular *N* = 83	Normed *N* = 83	Reversed *N* = 82	All^b^
	*N* (%)	*N* (%)	*N* (%)		*N* (%)	*N* (%)	*N* (%)	
Preferred format								
Plain	6 (9.8)	14 (22.2)	6 (9.0)	26 (13.6)	4 (5.9)	8 (4.1)	5 (8.2)	17 (8.5)
Lines with clinical significance	27 (44.3)	20 (31.7)	19 (28.4)	66 (34.6)	32 (47.1)	28 (38.9)	26 (42.6)	86 (42.8)
Lines with confidence limits	28 (45.9)	29 (46.0)	42 (62.7)	99 (51.8)	32 (47.1)	36 (50.0)	30 (49.2)	98 (48.8)
Missing	17	14	11	42	15	11	21	47

^a^ *p* = 0 < 001 for *χ* ^2^ goodness-of-fit test for equal distribution of responses (33%) for clinicians across formats

^b^ *p* = 0 < 001 for *Χ* ^2^ goodness-of-fit test for equal distribution of responses (33%) for researchers across formats

Qualitative comments, reflecting both support and lack of support for each of the formats, are tabulated in Table 5. Some respondents randomized to the “more” line type found the difference in direction of scoring for function versus symptom domains confusing, whereas others randomized to the “better” line type found higher symptom scores indicating improvement in symptoms to be counterintuitive. Some respondents valued the additional statistical information provided by confidence limits while some preferred asterisks alone. Others noted potential confusion regarding the use of asterisks to indicate clinical importance, since asterisks are also commonly used for statistical significance. Some respondents questioned how clinical importance was determined.

**Table 5:** Representative qualitative comments regarding interpretation and clarity of the graph formats

	Positive comments	Negative comments	Other insights
Comments on line-graph type			
“More” line type	As plain graphs these are quite clear [C01]^a^	They are somewhat confusing…whether it’s physical or fatigue is in one graph lower and in one graph higher…requires very close attention to detail [C04]	Regarding if higher is better or worse—need consistency [R]^b^
		It’s confusing whether lines going up or down should have negative or positive connotations because it’s mixed on the four graphs [C07]	They would be better if have the same direction (for example up = better) [R]
“Better” line type	Reviewing the graph, I understand the scale now and it was fairly simple to figure out [C09]	This one is more confusing in that severe fatigue is at the bottom as opposed to the top…my inclination would be that as fatigue worsens it would go up [C09]	It’s a bit unusual to have “reverse” scaling on pain and fatigue (i.e., lines going up means less pain or doing better) [R]
			The two lower graphs were harder to digest…I’d expect it was plotting pain level with higher on the y-axis reporting MORE pain [R]
“Normed” line type	The graphs are quite clear and descriptive [C06]	The contrast between treatments is clear, but the magnitude of the effect is absent [R]	
		No anchors on the Y axis makes it hard to tell if differences are meaningful [R]	
Comments on line-graph variations			
Plain graphs	The graphs themselves are quite clear [C01]	There is no confidence interval, there is no asterisk, so my only reference point was this little thing that says *P* = 0.01…it’s hard to know at what time points that was determined at [C02]	I like that the treatment is linked to the specific lines (versus a traditional legend) [R]
	They’re a little bit easier to read [C07]	[The other formats] offer more statistical information that is helpful to the clinician [C05]	What does the p value represent? You have repeated measures, is it the difference at the final time point? [R]
	The graphs are clear and if these were the only graphs provided to me I wouldn’t know what I was missing [C05]		
Indication of clinical significance	I believe the asterisk format is the easiest in showing patient results without the confidence intervals [C06]	The legend says that the difference is determined to be clinically important, although I don’t exactly understand what that means [C02]	Presumably, the method of determining a “clinically important difference” would be explained in the article text [R]
	The asterisks were actually helpful at the different months… because then I know if the differences were significant or not at that point in time [C07]	Using asterisk to denote clinical importance was confusing since it is often used in other studies to reflect statistical significance [R]	How am I supposed to determine clinically significant differences based on these graphs … What measure was used? Is there an established MCID? [R]
	The “clinically important difference” was indicated by an asterisk. That was helpful [R]	They’ve eliminated the confidence intervals so I don’t really have a sense of how statistically different everything is [C04]	The graph does not indicate how the lines were estimated/calculated and as a result no information how the p-values were calculated [R]
Indication of confidence limits	The asterisks and the demarcation of the confidence interval at every time point is very helpful [C05]	I think the confidence intervals plus the asterisks are redundant to see which time points are clinically significant...they were a little harder to read [C07]	The * and *p* value meanings differ, and the curves therefore highlight the ambiguity concerning statistical significance and clinically important differences [R]
	It gave both standard errors to give you an idea of the disbursement of the data as well as the asterisk to demonstrate statistical significance^c^ in the difference between them [C04]	There are a lot of lines and they’re overlapping so it’s a little bit less easy to interpret, so I think I like the simplicity of the (clinical significance) [C08]	May be difficult for some clinicians to interpret 95% confidence limits as currently explained [R]
	What’s also quite interesting is that there is a significant clinical change between these two treatments, however, the confidence intervals themselves overlap [C01]	While the error bars are a little bit helpful for understanding the spread of the data better, visually it gets a bit cumbersome [C09]	
Comments on formats showing proportions changed			
Pie charts	I think it’s just easier for my brain to see and compare the two charts…the bar graph takes me a little bit longer to compare the treatments [C08]	It’s not a format that I’m used to seeing to have the data presented and so it did catch me off guard initially [C09]	At 9 months Treatment Y had 50% improvement, Treatment X had 40% improvement, but the P value is 0.10 demonstrating non-significance. I put down treatments are about the same, but I’m confused as to what the *p* value truly means [C03]
	A pie chart is always easier on the eye [R]	Bar graph is easier to describe patient results compared to the pie graph [C06]	What I find ambiguous is that…because we have three variables for each comparison, we don’t know if we’re comparing improved versus improved or the ratio of improved to same to worsened [C01]
	The pie chart gives me percentages of patients stating improvement, worsening or about the same and I think that’s important information in discussions with the patient about treatment decision making [C05]	Please read any graphics design book that you can grab hold of …Never use pie charts! [R]	For the first question on physical function, the answer depended on whether an alpha of 0.05 or 0.10 was being considered. Since none was stated, I assumed the classical alpha = 0.05 [R]
Bar charts	(Bar charts)...can show each category, improved, about the same or worsened, head to head against the two treatments…for pie graphs you have to bounce back and forth to see the direct comparisons [C01]	The bar graph takes me a little bit longer to compare the two (treatments) [C08]	We’re given a test of significance but we don’t know what is being compared in that test [C01]
	Beauty is in the eye of the beholder I guess, but the bar graph…when you’re talking with patients I think clearly shows what you’re trying to describe [C06]	These bar charts are very confusing…there is no confidence interval at all…I don’t have a score to determine if patients improved how much did they improve; if patients worsened how much did they worsen [C03]	I think what’s confusing to me is that the *p* value is in the bottom right of each of the graphs, but it’s just unclear what was being compared [C02]
	I find this graph to be much easier to read than the pie charts [R]	I find these bar charts to be difficult to interpret. They take more time and likely are going to be more prone to error in interpretation [C05]	Technically the overall p-value that probably derives from a *Χ* ^2^ type of test cannot be used to justify statements about individual comparisons as the two statements were phrased…instead the comparative residuals of the expected comparison could be used to highlight where differences occur [R]

^a^Clinician comments are taken from the in-person interviews. The number in parentheses denotes the specific clinician providing the comment

^b^Representative researchers’ comments are taken from the online open-ended text boxes

^c^This respondent may have misinterpreted the meaning of the asterisks (clinical not statistical significance)

### Findings for formats illustrating proportions changed

**Fig. 6:**
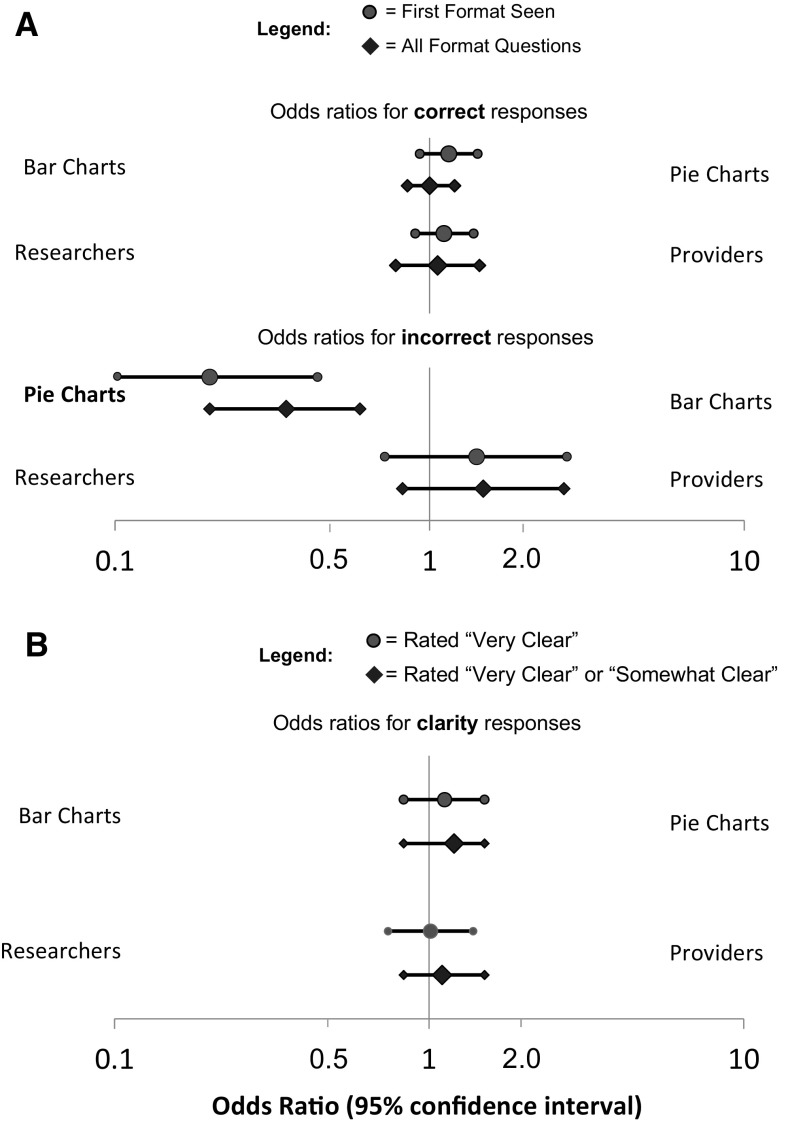
Multivariable modeling results displayed as odds ratios for accuracy and clarity ratings for proportions changed formats. For interpretation please see Fig. 5 legend

Regarding accuracy of interpreting formats illustrating proportions changed, clinicians who saw pie charts were more likely to respond correctly to the first two accuracy questions than those who saw bar graphs (20 vs. 17%), though the opposite was true for researchers (7% for pie charts vs. 15% for bar graphs), see Table 6. The most common response was selection of the treatment that was better in absolute terms, rather than “about the same,” which was deemed to be the correct answer given that the between-treatment differences were not statistically significant as indicated by the *p* value (Fig. 4). With regard to selecting the wrong (incorrect) treatment, researchers and clinicians were less likely to select the incorrect treatment when pie charts were displayed (5.1 and 1.6%, respectively) compared to bar charts (14.7 and 11.2%, respectively). Multivariable modeling analyses showed that the odds of selecting the “correct” response did not differ significantly by chart format or by respondent type, whereas the odds of selecting the “incorrect” treatment were significantly lower with the pie charts (OR 0.35; 95% CI 0.2–0.6; *p* < 0.001) but did not significantly differ by respondent type (OR 1.48; 95% CI 0.82–2.67; *p* = 0.198, Table 3). Complete descriptive results are listed in Table 6, and the modeling results are displayed graphically in Fig. 6.

**Table 6:** Accuracy and clarity responses for “proportions changed” formats

	Clinicians				Researchers			
	Pie charts *N* = 117		Bar charts *N* = 116		Pie charts *N* = 123		Bar charts *N* = 125	
	*N*	(%)	*N*	(%)	*N*	(%)	*N*	(%)
Accuracy: first format seen								
Physical activities (function domain)								
Treatment X	0	(0)	4	(3.4)	0	(0)	2	(1.6)
Treatment Y	69	(59.0)	69	(59.5)	95	(77.2)	70	(56.0)
About the same^a^	31	(26.5)	27	(23.3)	13	(10.6)	29	(23.2)
Missing	17	(14.5)	16	(13.8)	15	(12.2)	24	(19.2)
Pain (symptom domain)								
Treatment X^a^	87	(74.4)	80	(69)	101	(82.1)	80	(64.0)
Treatment Y	6	(5.1)	15	(12.9)	2	(1.6)	16	(12.8)
About the same	7	(6.0)	5	(4.3)	5	(4.1)	5	(4.0)
Missing	17	(14.5)	16	(13.8)	15	(12.2)	24	(19.2)
Number of questions correct								
Both questions	23	(19.7)	20	(17.2)	8	(6.5)	19	(15.2)
One question	72	(61.5)	67	(57.8)	98	(79.7)	71	(56.8)
Neither question	22	(18.8)	29	(25.0)	17	(13.8)	35	(28.0)
Number of questions incorrect^b^								
Both questions	0	(0)	1	(0.9)	0	(0)	2	(1.6)
One question	6	(5.1)	17	(14.7)	2	(1.6)	14	(11.2)
Neither question	111	(94.9)	98	(84.5)	121	(98.4)	109	(87.2)
Clarity rating: first format seen								
Very clear	85	(42.5)	71	(35.5)	80	(38.1)	76	(36.4)
Somewhat clear	63	(31.5)	68	(34)	70	(33.3)	73	(34.9)
Somewhat confusing	37	(18.5)	48	(24)	49	(23.3)	51	(24.4)
Very confusing	15	(7.5)	13	(6.5)	11	(5.2)	9	(4.3)
Missing	33		33		38		39	
Format preference								
Format preferred	99	(50.0)	98	(50.0)	90	(44.3)	113	(55.7)
Missing	36				45			

^a^Denotes “correct” response

^b^Answers were coded as incorrect when the wrong treatment was selected by the respondent (answers to “about the same” response were ignored—see text for further details)

Regarding format clarity ratings, 74% of clinicians and 71% of researchers rated pie charts “somewhat” or “very” clear, and 70% of clinicians and 71% of researchers rated bar graphs “somewhat” or “very” clear. These differences were not statistically significant in the multivariate models, when testing for an association with chart format or for an association with responder type (Table 3). In terms of their preferences, more respondents overall preferred bar charts (52.8%) over pie charts (47.2%, *p* = 0.03); clinicians were equally split between bar and pie formats (50 vs. 50%) but more researchers preferred bar charts (56% bar vs. 44% pie charts, *p* = 0.11).

Qualitative themes coded from the one-on-one interviews and online comments are summarized in Table 5. Illustrative comments reveal that several respondents were negative about pie charts, whereas others were negative about bar charts, each for different reasons. Further, comments concerning the p-value annotations reflected uncertainty about the meaning of p = 0.10 for the physical domain, as well as uncertainty about how to clinically interpret a significant p-value below 0.05.

## Discussion

With the increasing prevalence of PROs in clinical trials and other comparative research studies, information on how to present PRO findings so that clinicians can understand them and incorporate them into clinical practice is critical. In previous research, clinicians often expressed difficulty interpreting PROs, [11, 20] so research evaluating clinicians and researchers’ accuracy of interpretation, and presentation factors associated with these interpretations is critically needed [21]. Our survey of clinicians and researchers showed that differences in displays of longitudinal PRO data were associated with differences in how accurately they were interpreted and on how clear they were rated to be.

A key finding is that clinicians and PRO researchers sometimes misinterpreted simple graphs of PRO scores when displayed in an array of function and symptom domains. This misinterpretation resulted in the incorrect treatment being chosen as superior for PROs, and was consistently observed across formats for both clinicians and researchers, suggesting a need to report PRO findings as clearly as possible. For graphing mean PRO study results over time, scale direction impacted on the misinterpretation rate, as did presenting normed data. Further, clarity ratings were also higher when directionality was consistent (higher = better results). Annotations of line graphs to illustrate clinical or statistical significance were favored by the majority of respondents, and did not decrease interpretation accuracy. For displays of proportions of patients changed from baseline, misinterpretation occurred with both pie charts and bar charts and among both researchers and clinicians (although no differences were detected between the two responder groups).

These findings should be interpreted in the context of the study design and its limitations.

The internet survey relied on convenience samples using online platforms, and on self-reported eligibility. Although a variety of target populations were identified, a snow-ball sampling approach can lead to a less diverse sample as individuals tend to recruit others like themselves. The overall sample size was sufficiently powered to detect statistically significant results, but provided limited power for subgroup analyses. Further, we did not ascertain how familiar respondents were with the presentation formats, and learning effects may have affected the findings. Accuracy of interpretation required attention to the displayed p-value (but analysis of “incorrect” responses overcame this limitation). We limited the scope of presentation issues to representation of the trial PRO results, and did not include additional information such as compliance tables or number of patients providing data at given time points; further research is required to address these issues. Strengths of the study include the large sample resulting from online distribution, thus including participants from a variety of locations. The survey design including 18 versions to control for order effects improved the robustness of the findings. The one-on-one interviews permitted purposive sampling and provided qualitative data that supplemented the online comments and complemented our previous qualitative findings [14, 15].

Improving the accuracy of interpretation of PRO study findings remains a challenge for the field in many ways. First, using graphic presentations that most clinicians and researchers intuitively understand may help, and findings from the present study could inform the selection of optimal data presentations. Second, our quantitative and qualitative findings make clear that no one approach for either longitudinal data or proportions changed is universally appealing, nor is free of misinterpretation errors. Thus, additional strategies beyond design of presentation are required. Third, consistency of PRO presentation is very likely to have value. In oncology for example, survival curves and toxicity data from randomized clinical trials are typically reported consistently across trials, whereas PRO data reporting is highly variable. Fourth, our qualitative findings suggest that challenges in interpreting constructs that underlie the data—such as the nature of cut-points used to categorize patients into proportions changed, or the meaning of a particular numeric group score—also contribute to the perceived clarity and understanding of PRO displays. Our findings suggest that adding annotations such as indications of score meaning and clinical significance may assist with clinical interpretation.

In conclusion, this study with its large sample, combination of quantitative and qualitative data, and careful design can inform best practices for presentation of PRO data to improve interpretability. No presentation formats were free of interpretation error; however, respondents were less likely to make interpretation errors when mean group data were not normed, and when proportions changed were displayed in pie charts. These results can inform best practices for displaying of PRO data.

## Electronic supplementary material

Below is the link to the electronic supplementary material.

Supplementary material 1 (DOCX 20 KB)
