# Supplementary material for: Presenting comparative study PRO results to clinicians and researchers: beyond the eye of the beholder
Source: Qual Life Res. 2017 Nov 2;27(1):75–90. doi: 10.1007/s11136-017-1710-6 (PMC5770492; doi:10.1007/s11136-017-1710-6)
Supplement: Supplementary file 1 — Supplementary material 1 (DOCX 20 KB) [file 11136_2017_1710_MOESM1_ESM.docx]

**Table S1: Overview of Survey Version Order**

|  | **Line Graphs of Average Scores** | | | **Proportions Changed** | |
| --- | --- | --- | --- | --- | --- |
|  | **Format 1** | **Format 2** | **Format 3** | **Format 4** | **Format 5** |
| **Version 1:**  **“More” Format** | **Confidence Limits** | **Clinical Significance** | **Plain** | **Pies** | **Bars** |
| **Version 2**  **“More” Format** | **Plain** | **Confidence Limits** | **Clinical Significance** | **Pies** | **Bars** |
| **Version 3**  **“More” Format** | **Clinical Significance** | **Plain** | **Confidence Limits** | **Pies** | **Bars** |
| **Ver. 4-6**  **“Normed” Format** | **As per 1-3, lines normed to 50** | | | **Pies** | **Bars** |
| **Ver. 6-9**  **“Better” Format** | **As per 1-3, Higher scores always indicated ”better” outcome** | | | **Pies** | **Bars** |
| **Ver. 10-18** | **As per 1-9** | | | **Bars** | **Pies** |
